# Are Sport and Exercise Science Students Receiving Enough Training to Adequately Design Exercise Programs for Patients with Cancer, Overweight, or Obesity in Spain? A Comprehensive Archival and Survey Analysis of 52 Spanish Universities

**DOI:** 10.1007/s13187-025-02580-8

**Published:** 2025-02-13

**Authors:** Alejandro Gómez-Bruton, Alex Gónzalez-Agüero, Gabriel Lozano-Berges, Angel Matute-Llorente, Nuria Garatachea, German Vicente-Rodríguez, Jose A. Casajús

**Affiliations:** 1 EXER-GENUD (EXERCISE-Growth, Exercise, NUtrition and Development) Grupo de Investigación, Universidad de Zaragoza, Saragossa, Spain; 2 Facultad de Ciencias de La Salud y del Deporte, Departamento de Fisiatría y Enfermería, Universidad de Zaragoza, Huesca, Spain; 3 Centro de Investigación Biomédica en Red de Fisiopatología de La Obesidad y Nutrición (CIBEROBN), Instituto de Salud Carlos III, Madrid, Spain; 4 Instituto Agroalimentario de Aragón (IA2), Universidad de Zaragoza, 50009 Saragossa, Spain; 5 Facultad de Medicina, Departamento de Fisiatría y Enfermería, Universidad de Zaragoza, Saragossa, Spain

**Keywords:** Oncology, Education, University, Training, Lecturing, Obesity

## Abstract

Obesity and cancer are two of the most significant global public health concerns worldwide. Exercise in preventing and managing these diseases has become a prominent area of research, and BSc sport and exercise science (SES) graduates are among the professionals involved in designing exercise programs for these populations. The aims of the present study were (1) to quantify the number of teaching hours focused on exercise in patients with cancer or overweight/obesity and (2) to collect perceptions of SES teachers of whether these hours are enough to prescribe exercise to these populations adequately. An online survey was sent to 97 university teachers from 58 different institutions in Spain offering the sport and exercise sciences degree. The survey included questions designed to ascertain the number of hours lectured on the topics of exercise and cancer or overweight/obesity. Additionally, it aimed to gather teachers’ perceptions of how prepared students were to work with these populations. Seventy-six teachers (representing a 78% response rate) from 52 different centers (representing 88% of the centers) completed the survey. The teachers reported an average of 8.7 class hours dedicated to exercise and cancer and 17.1 class hours dedicated to exercise in patients with overweight/obesity. Teachers expressed low satisfaction with the number of class hours allocated to cancer education (2.2 points out of 5) and a neutral response regarding the allocation of hours to overweight/obesity (3.1 points out of 5). The findings of the current study suggest that Spanish SES students may be receiving a limited amount of instruction in the area of exercise prescription for patients with cancer and overweight/obesity. Although the curriculum is explicitly dedicated to training SES students, teachers believe that additional training in specific exercise prescription for people with cancer would enhance SES qualifications.

## Introduction

Obesity and cancer represent two of the most significant global public health concerns worldwide, exhibiting alarming prevalences that continue to increase and carry a substantial economic burden [1, 2]. The World Health Organization (WHO) estimates that in 2022, more than 2.5 billion adults were overweight, with over 890 million of these individuals classified as obese [3]. Concurrently, cancer persists as a leading cause of mortality, with 19.3 million new cases and nearly 10 million deaths in 2020 [1]. The relationship between obesity and cancer is well documented, with approximately 4 to 8% of all cancers attributed to obesity and an increased risk of mortality in patients with obesity who suffer from cancer [4]. These statistics underscore the urgency of effectively intervening in these health problems.

There is a substantial body of scientific evidence to suggest that regular physical activity and exercise can reduce the risk of developing certain types of cancer [5] and improve survival in cancer patients [6]. Moreover, physical activity and exercise have been demonstrated to reduce treatment side effects and improve the quality of life and cancer-related fatigue [7]. Consequently, increasing daily physical activity and improving exercise prescription represent crucial elements in the management and prevention of these diseases.

Sport and exercise sciences (SES) graduates should develop and oversee the implementation of exercise programs for individuals who are overweight or obese, as well as those who have been diagnosed with cancer. Their training encompasses a comprehensive understanding of exercise prescription, physiology, and biomechanics, enabling them to develop tailored exercise programs adapted to each patient’s particular needs and preferences. In certain countries, like Australia, specifically trained SES graduates, referred to as accredited exercise physiologists, are integrated into the healthcare system, providing indispensable services in hospitals and clinics [8]. However, in other countries such as Spain, these professionals are not included at a state level within the health system, which limits access to these specialized services.

This may change in the near future, as organizations such as the Spanish Society of Medical Oncology (SEOM) have recently developed a referral pathway that includes SES graduates as part of an interdisciplinary team for the management of cancer patient [9]. In the same line, the Spanish Society for the Study of Obesity (SEEDO) has developed a guide focusing on strategies to promote physical activity to prevent obesity, with one strategy focusing on incorporating physical activity into the national health system and improving the training of SES graduates working with patients with obesity [10]. This is in line with the consensus joint document of the SEEDO and the Spanish Society of Primary Care Physicians (SEMERGEN) which stated that the role of the SES graduate would be of paramount importance in the planning of specific exercise programs [11].

It is, therefore, imperative to establish the baseline level of training of SES graduates when they are tasked with designing specific interventions for patients with overweight, obesity, and/or cancer in Spain to ascertain if extra training is needed before working with the aforementioned populations. Thus, the aims of the present study were (1) to quantify the number of teaching hours allocated to specific training focused on patients with cancer or overweight/obesity in all the Spanish universities that offer the SES degree during their undergraduate 4-year programs and (2) to register teachers perceptions of whether the number of hours received by each student is adequate to correctly prescribe exercise to these populations.

## Methods

### Study Design

This is a cross-sectional study that firstly used an archival design relying on existing data to collect information from all the universities that offer the degree of SES in Spain. After collecting the necessary information, a survey was sent to the coordinator of the subjects/courses that may lecture about exercise in patients with cancer or overweight/obesity as explained below. The processing of data was performed in compliance with the provisions of Regulation (EU) 2016/679, General Data Protection Regulation (RGPD) and Organic Law 3/2018, on the Protection of Personal Data and Guarantee of Digital Rights (LOPDyGDD) and was approved by the Data Protection Delegate of the University of Zaragoza (RAT 2024–167).

### Data Collection

AGB first developed the data extraction process using an archival design relying on existing data to collect information from all the universities offering the degree of SES in Spain. After collecting the necessary information, a survey was sent to the coordinator of the subjects/courses that may lecture about exercise in patients with cancer or overweight/obesity, in three consecutive stages.

The initial step involved accessing the Spanish Ministry of Science, Innovation and Universities website and opening the specific tab, “QEDU: What to study and where?” to search for SES degrees. A list of 67 public and private centers offering the degree of SES was obtained. An Excel sheet was developed from that list, including the universities that offered the degree.

Secondly, a systematic web page search of all curricular guide programs of each Spanish SES degree was undertaken, and the specific study plan (comprising all the degree courses) was evaluated. Courses related to the following areas were sought:

- Physical activity and health
- Physical activity and special populations
- Physical activity and disease
- Physical activity and exercise prescription

Third, when a course that could match the previous description was identified, it was recorded in the Excel sheet, and the curricular academic guide (a document that outlines the course objectives, content, evaluation methods, etc.) was downloaded. The contents section was then searched for the following terms:

- Oncology
- Cancer
- Overweight
- Obesity
- Other diseases, pathologies or special populations^1^

The following data were registered in the Excel sheet: mention of cancer/oncology (YES/NO), mention of overweight or obesity (YES/NO), and mention of other populations that could include cancer, overweight or obesity (YES/NO). If one of the three items was answered with a YES, the teacher´s details (name and email) were recorded, and a survey containing the questions listed in Table 1 was sent.

**Table 1:** The questionnaire sent to teachers of the courses that could include content focusing on exercise or physical activity in people with cancer, overweight, or obesity

1	University name
2	Name of the course you lecture
3	Annually hours of lectures (theory lessons) you dedicate to exercise and people with cancer
4	Annually hours of lectures (theory lessons) you dedicate to exercise and people with overweight or obesity
5	Annually hours of practical lessons you dedicate to exercise and people with cancer
6	Annually hours of practical lessons you dedicate to exercise and people with overweight or obesity
7	Annually hours of seminars or problem solving you dedicate to exercise and people with cancer
8	Annually hours of seminars or problem solving you dedicate to exercise and people with overweight or obesity
9	Do you consider that the received classes are sufficient to adequately prescribe exercise for people with cancer?
10	Do you consider that the received classes are sufficient to adequately prescribe exercise for people with overweight or obesity?
11	Does your degree include any other courses that include contents focusing on exercise with people with cancer, overweight or obesity?
12	Does your university offer a master degree focused on exercise assessment/prescription in people with cancer?
13	Does your university offer a master focused on exercise assessment/prescription in people with overweight or obesity?

Questions 3 to 8 were presented with a scale ranging from 0 to > 20 h

Questions 9 and 10 consisted of the following Likert scale: (1) totally disagree, (2) disagree, (3) neutral (I don’t agree nor disagree), (4) agree, (5) totally agree

If a university did not include any compulsory or optional courses in which the aforementioned five medical terms were included in the content section of the course, an email was sent to the coordinator of the course “Exercise and Health” (or a similar course if it was not offered) to obtain at least one response from each university and so that the teacher could alert the researchers if there were another unit/course that had not been detected by answering question 11. The survey was created and completed through Google Forms and was open from 10 July 2024 to 20 September 2024. A friendly reminder was sent the first week of September to all coordinators who had not replied to the questionnaire to improve the response rate.

### Data Curation and Analysis

An Excel sheet was downloaded from Google Forms that included all the responses provided by the contacted teachers. If a teacher had completed two surveys because he/she was the coordinator of two different courses related to the topic, both surveys were kept. Nonetheless, data for questions 9 and 10 were deleted from one survey to avoid having duplicate data (two equal responses from one teacher). The categories (totally disagree, disagree…) from questions 9 and 10 were then transformed into numbers (1 to 5). When teachers responded > 20 h in questions 3 to 9, these were transformed to 21 h (this was the case for three responses). Means and standard errors were calculated when appropriate with Excel sheets.

## Results

### Included Universities

The initial search in the Spanish Ministry of Science returned a total of 58 universities (both public and private) that had 67 centers offering the SES degree (some universities had the same degree taught in 2 or 3 different centers). From these, the Universidad Europea de Valencia did not offer the degree in the academic year 2023–2024. The Universidad de la Laguna started the degree in the academic year 2022–2023, but they had not begun to offer the courses related to health and exercise (they were optional courses offered in the last year 2025–2026). Two degrees were going to start in the year 2024–2025 (Universidad de las Illes Balears and Universitat Abat Oliba CEU), and one degree was not a SES degree (Euncet Business School: Universidad Politécnica de Catalunya).

Additionally, the QEDU webpage suggested that the Universidad Pontificia de Salamanca offered two SES degrees, but the researchers found only one. The Universidad de Girona and the Universidad Rovira i Virgili each offered two SES degrees in affiliated centers that were common for both (EUSES Campus Salt and EUSES Campus Deltebre).

An additional campus was identified in the Universidad CEU Fernando III, but this center was a new one that was going to substitute another university (CEU Cardenal Spinola) and was therefore not included in our analysis.

Therefore, from the 67 centers eligible, 59 centers were screened.

### Response Rate

**Fig. 1:**
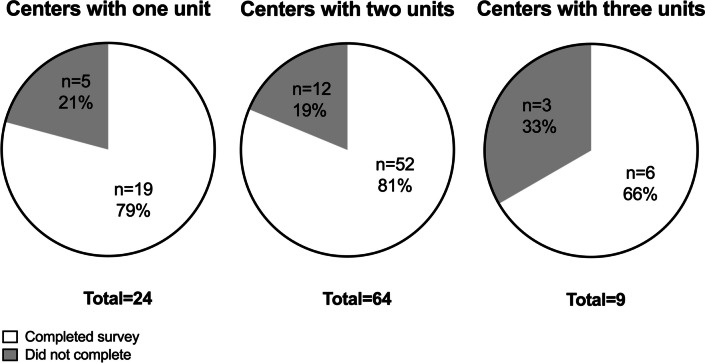
Response rate distributed by the number of units/courses providing content on exercise prescription in patients with overweight/obesity or cancer

A total of 92 emails were sent to the course´s coordinators in the first wave. From the received answers, five new units/courses were identified, and the corresponding coordinators were contacted making a total of 97 sent emails. A total of 76 surveys were completed, representing a 78% response rate. From the 59 contacted centers, 52 (88%) completed at least one survey. Figure 1 displays the global response rate divided according to the number of units/courses each center offers focusing on the topic of interest.

### Number of Hours Focused on Physical Activity and Overweight/Obesity or Cancer

**Fig. 2:**
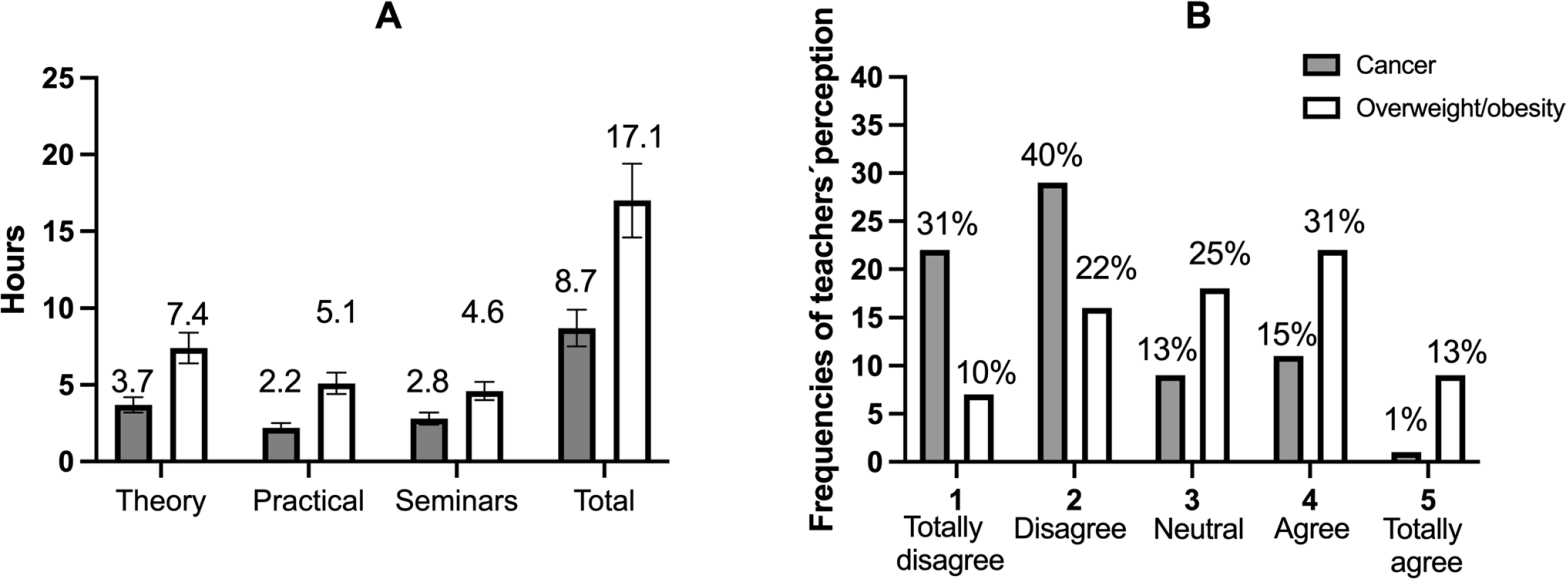
(2**A**) The mean and standard error for the different types of classes and the total number of hours that SES students receive when they finish their degree, and (2**B**) the frequencies and percentages of teachers’ perception regarding the preparation of their students to adequately prescribe exercise in patients with cancer or overweight/obesity (scale 1 (totally disagree) to 5 (totally agree))

Teachers from 52 universities reported an average of 17 h of lecturing focused on exercise and overweight/obesity during the SES degree. As shown in Fig. 2A, the majority of the teaching load consisted of theoretical classes (7.4 h) followed by practical classes (5.1 h) and seminars (4.6 h).

The number of hours was lower for exercise and cancer-related topics, with an average of 8.7 h along the SES degree, which were distributed among theory (3.7 h), seminars (2.8 h), and practical lessons (2.2 h).

### Available Higher Education-Specific Courses

When asked about higher education courses (master degrees or specialization degrees) offered by their university to improve exercise assessment or prescription skills in people with overweight, obesity, or cancer, 25 teachers (33%) from 19 different universities answered positively to both cancer (question 12) and overweight/obesity (question 13).

### Teachers Perception

The average agreement of the teachers to the question “Do you consider that the received classes are sufficient to adequately prescribe exercise for people with cancer?” was 2.2 out of 5. For the question, “Do you consider that the received classes are sufficient to adequately prescribe exercise for people with overweight/obesity?” the average response was 3.1 out of 5. As illustrated in Fig. 2B, on a 1 (totally disagree) to 5 (totally agree) scale, most teachers rated with a 2 and a 1 when asked about exercise prescription for people with cancer and with a 4 and a 3 when asked about exercise prescription for people with overweight/obesity.

## Discussion

The present study is the first to investigate the amount of lecture time that SES Spanish students receive, focusing on overweight, obesity, or cancer. The findings indicate that increasing the number of hours dedicated to these subjects in Spanish SES degrees could lead to better specific preparation for exercise prescription in these populations. Spanish SES educators rated their satisfaction with the current teaching hours in the SES degrees at an average of 2.2 points for exercise prescription for people with cancer and 3.1 points for people with overweight or obesity (on a scale of 1 to 5).

SES educators of the involved universities reported an average of 8.7 teaching hours focused on specific exercise prescription or assessment in patients with cancer. This seems like a very low volume of hours allocated to this population, especially considering that different types of cancer might need different types of treatment and exercise prescriptions.

The low amount of classes/courses dedicated to this population in Spanish SES degrees is similar to that reported in a previous study developed in Italian universities which showed that only 39% of the students reported the presence of courses that explained exercise prescription for patients with cancer before surgery while 36% and 44% of the students reported that their universities offered courses during or after therapy conclusion, respectively [12]. A comparison with other SES BSc students from other countries was not possible due to the lack of available studies.

When comparing our results with those of other health professionals, such as physicians, a study evaluating doctors of medicine and osteopathic medicine institutions in the USA found that 51.5% did not offer courses related to general physical activity. Moreover, in those universities that did offer related courses in 82.2% of the cases, these were elective [13]. In the same line, we found in a previous study that 94.3% of Spanish medical school students demanded more education in physical activity, as only 34.1% felt prepared to recommend physical activity [14]. Similar results were found when assessing family medicine residents’ knowledge, competence, and perspectives of exercise prescription in Canada, as only 14.9% perceived their training was adequate to correctly prescribe exercise and 91% desired more training. Other professionals that might have to work with patients with cancer or overweight/obesity are nurses, but in Spain a study showed that only 5.82% of the nursing teaching credits are related to physical activity contents. Most of the contents were related to physical activity promotion, with the contents related to exercise prescription being neglectable [15]. Regarding physiotherapists, similar results were found in a multi-national survey assessing preparedness to prescribe physical activity and exercise to people with musculoskeletal pain which found that only 11 and 16% could name an accepted guideline for aerobic and resistance exercise, respectively, and only 38% had received training to deliver aerobic exercise and 50% to deliver resistance training [16]. To the best of our knowledge, to date, no studies have evaluated the education that physiotherapists receive in Spain focusing on exercise for people with overweight/obesity or cancer. Nevertheless, Spanish study plans are designed following the Spanish Ministry of Science and Innovation guidelines (ORDEN CIN/2135/2008), which do not include the words “exercise” or “physical activity.” It would therefore be expected that a lower amount of hours of exercise prescription in these pathologies would be found in the physiotherapy academic study plan and further studies similar to this one should be performed to ascertain the level of formation in physiotherapy students in these populations.

It can be reasonably deduced that the majority of health professionals engaged in the field of exercise prescription in Spain may require supplementary training upon completion of their BSc degree to adequately prescribe exercise to people with cancer. This additional training is available in Spain, as evidenced by the fact that 19 different MSc were mentioned by the surveyed teachers, all of which encompass content related to fitness assessment or exercise prescription in individuals with cancer or overweight/obesity.

SES students received more hours of exercise in patients with overweight/obesity with teachers reporting an average of 17.1 h of classes during the SES degree. This was accompanied by higher punctuation in the question “Do you consider that the received classes are sufficient to adequately prescribe exercise for individuals with overweight/obesity?” with an average punctuation of 3.1, which is closest to the “Neutral (I don’t agree nor disagree)” option of the survey. SES students should receive adequate teaching about this condition, as previous studies have shown that SES students exhibit moderate fat phobia and endorse specific anti-fat stereotypes [17]. In fact, the aforementioned study showed that those SES students with an internalization of the athletic ideal may blame overweight and obese people for their condition and believe that being fat is not an acceptable body shape and size. These students would benefit from significantly increasing the number of lectures and practical sessions they undertake focusing on this population. This would enable them to alter their perceptions about people with obesity. Furthermore, other conditions, such as type 2 diabetes and metabolic syndrome, are strongly linked to obesity. Consequently, improving students’ knowledge and skills in working with people with obesity will also enhance their ability to work with people with other cardiometabolic conditions.

Although the number of specific hours dedicated to the focus areas of cancer and overweight/obesity by SES students may appear limited, the global amount of hours dedicated to these populations is likely higher due to (1) SES Spanish students are required to complete mandatory courses that focus on fitness assessment and exercise prescription in the general population, as well as in older adults and other populations not included in the present study. These courses could potentially teach transversal skills to the involved students, including the ability to conduct cardiorespiratory assessments through direct and indirect tests, evaluate physical activity through questionnaires or accelerometry, calculate basal metabolic rate and total energy expenditure, and assess sedentary behaviors. Such skills and knowledge could later be applied to patients with cancer or overweight/obesity. (2) The completed survey exclusively addressed the issue of face-to-face teaching hours. It is possible that teachers may assign tasks related to cancer or obesity that students would have to complete at home. This would have the benefit of improving their skills and knowledge in these areas. (3) The survey had an option of > 20 h that was coded as 21 h. This amount of hours represents the minimum of that category and could underestimate the volume of hours that SES students receive (although this was only the case for three universities).

It may be beneficial for Spanish universities offering the SES degree to consider adapting their teaching itineraries to include a greater focus on prescription courses for patients with cancer. This would benefit the students and, more importantly, the final patients receiving the exercise interventions. If this is not feasible, universities should explore the possibility of offering additional courses or diplomas that undergraduate students can undertake while completing their final years of the SES degree to enhance their knowledge and skills in these populations. This approach has been demonstrated to result in positive outcomes, as evidenced by student knowledge and skills improvements [18].

## Conclusions

The findings of the present study indicate that although SES students receive specific training in exercise prescription for patients with cancer and/or overweight/obesity, educators perceive that additional teaching hours are needed to improve exercise prescription in these populations, especially in cancer. A multitude of postgraduate programs exist in Spain that could facilitate the acquisition of these skills in future exercise professionals.

## Data Availability

The data that support the findings of this study are available from the corresponding author (AGB), upon reasonable request.

## Footnotes

1 Some academic guides specified several diseases/conditions (diabetes, arthritis, etc.) and dedicated a block of the course to exercise and other diseases/pathologies (which could include cancer or obesity) or special populations (but did not specify the populations).
